# Niacin-Induced Syncope in a Middle-Aged Male: When an Over-the-Counter Vitamin Goes Wrong

**DOI:** 10.7759/cureus.50454

**Published:** 2023-12-13

**Authors:** Jiannan Huang, Ibrahim Ahmed, Ayodeji Balogun, Khizar Hamid

**Affiliations:** 1 Internal Medicine, University of South Dakota Sanford School of Medicine, Sioux Falls, USA; 2 Hospital Medicine, University of South Dakota Sanford Medical Center, Sioux Falls, USA; 3 Pulmonary Disease and Critical Care Medicine, Corewell Health East, Royal Oak, USA

**Keywords:** hypercholesterolemia, syncope, nutritional supplement, otc vitamin, niacin

## Abstract

Niacin is an essential vitamin with lipid-modifying properties. It is readily available in many over-the-counter (OTC) supplements. However, the use of niacin can lead to undesirable adverse reactions, including flushing, nausea, hyperglycemia, etc.

Here, we present a rare case of niacin-induced syncope caused by a sudden increase in dosage in a middle-aged male. Extensive history, examination, and cardiovascular investigation were obtained to rule out various common etiologies of syncope. We also discuss the utility of niacin as a nutritional supplement, as most individuals obtain sufficient niacin intake from foods and beverages. As a treatment for dyslipidemia, niacin no longer exhibits cardiovascular benefits in the contemporary statin era. We argue that an additional niacin supplement is both unnecessary and potentially harmful. Therefore, niacin supplementation should be cautiously taken with no additional health benefits and frequent deleterious effects.

## Introduction

Niacin, also known as vitamin B3, is prevalently available in several over-the-counter (OTC) products such as multivitamin-mineral complex, B-complex vitamins, and niacin-only dietary supplements. Niacin is an essential human nutrient and its deficiency causes pellagra, an uncommon condition in industrialized countries. Niacin, in a high dose (over 3000 mg/day), is also a potent lipid-modifying agent that raises high-density lipoprotein cholesterol levels [1] with cardiovascular and mortality benefits in the pre-statin era [2]. However, contemporary evidence has pointed out the lack of benefit of niacin in patients receiving standard lipid-lowering treatment such as statins.

The toxicity profile of niacin is prominent. Some well-known adverse reactions of niacin include flushing, gastrointestinal discomfort, and hyperglycemia [3]. However, frank syncope induced by niacin is rarely described in the literature [4]. In this study, we present a patient with syncope after ingesting high-dose niacin and urge clinicians to recognize the net clinical harm of niacin.

## Case presentation

A male patient in his 50s presented to the emergency room (ER) with transient loss of consciousness. The patient routinely took one capsule of OTC 100 mg extended-release niacin daily for cholesterol reduction but switched to a 500-mg immediate-release formulation on the day of symptom onset. Thirty minutes after taking the higher dose of niacin, he experienced flushing in his face, chest, and bilateral upper extremities while resting on a couch. He had previous experiences of flushing with niacin but complained of substantially more intense flushing during the ongoing episode. He did not drink alcohol, exercise, or take a bath or shower before the onset of symptoms. Additionally, there was no history of sweating, coughing, sneezing, laughing, or strong emotional changes.

The patient subsequently developed lightheadedness and lost consciousness with urinary incontinence. A family member witnessed the presentation but did not report tongue biting or tonic/clonic movement. The patient regained full alertness after one minute with no residual confusion. Emergency medical services were called, and the patient was transported to the ER. During transportation, he complained of 6/10 substernal, non-radiating, pressure-like chest pain for 30 minutes. A review of systems revealed no other abnormality. The patient’s past medical history included hypertension and gastroesophageal reflux disease, and his home medications included lisinopril, multivitamin capsule, niacin, and as-needed pantoprazole.

Upon arrival at the hospital, the patient’s blood pressure was 115/73 mmHg, heart rate was 64 beats per minute, and oxygen saturation was 96% in room air. His orthostatic blood pressure was 117/68 mmHg, and he exhibited no signs of hypovolemia. His pupillary light reflex and extraocular movements were intact. There was no nystagmus, tongue deviation, sensory loss, extremity weakness, or abnormal deep tendon reflexes. There was no murmur on neck vasculature auscultation. The cardiopulmonary auscultation and the rest of the physical exam were nonrevealing.

**Figure 1:**
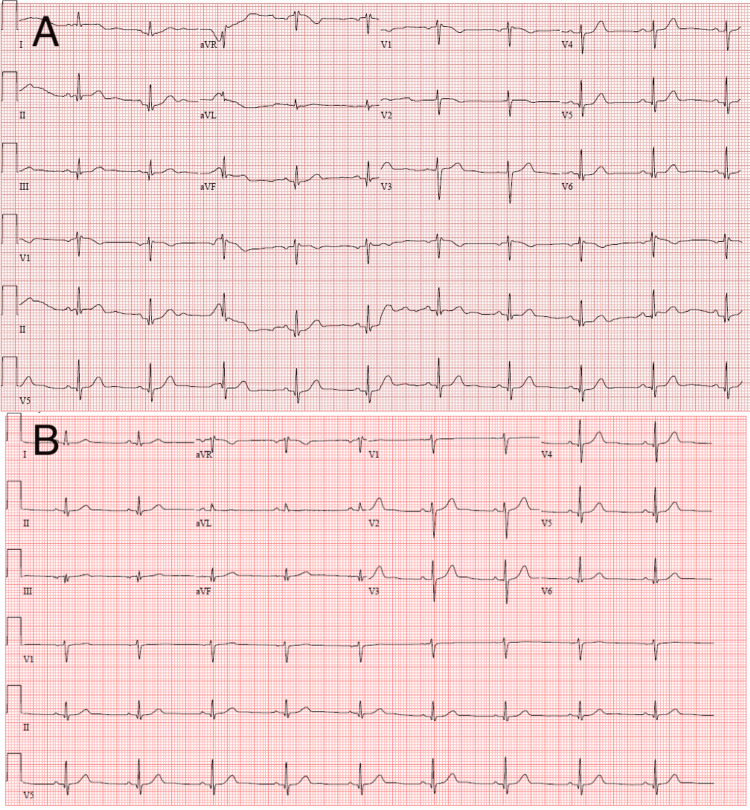
Electrocardiography (ECG) on admission (A) and from two years ago (B) ECG on admission showed sinus rhythm, RSR' in V1, and non-specific T abnormalities in aVL (A). There were no significant changes from the baseline ECG two years ago (B).

Laboratory investigations revealed a mild elevation of plasma troponin I, hypokalemia, and elevated plasma thyroid-stimulating hormone (TSH) levels (Table 1). Electrocardiography (ECG) (Figure 1) showed normal sinus rhythm without acute ST-T changes, PR/QRS/QT interval abnormality, or other patterns suggesting arrhythmias. A non-contrast computed tomography of the head showed no acute intracranial pathology. The patient’s symptoms resolved while in the ED, and he was admitted for syncope evaluation.

**Table 1:** Laboratory investigations of the patient on admission WBC; white blood cells, TSH; thyroid-stimulating hormone, ALT; alanine transaminase, HDL; high-density lipoproteins, LDL; low-density lipoproteins

Test Name	Result	Normal Range
Troponin I (ng/mL)	0.188	0.000-0.033
WBC count (K/uL)	4.9	4.0-11.0
Hemoglobin (g/dL)	14.6	13.5-17.5
Platelet count (K/uL)	144	140-400
Potassium (mq/L)	3.2	3.5-5.1
Magnesium (mg/dL)	2.0	1.6-2.6
Creatinine (mg/dL)	1.09	0.73-1.18
TSH (uIU/mL)	6.78	0.35-4.94
Free T4 (ng/dL)	1.1	0.7-1.5
ALT (U/L)	29	0-5
Total cholesterol (mg/dL)	123	<200
HDL cholesterol (mg/dL)	44	≥40
LDL cholesterol (mg/dL)	64	≤100
Triglyceride (mg/dL)	77	<150

**Figure 2:**
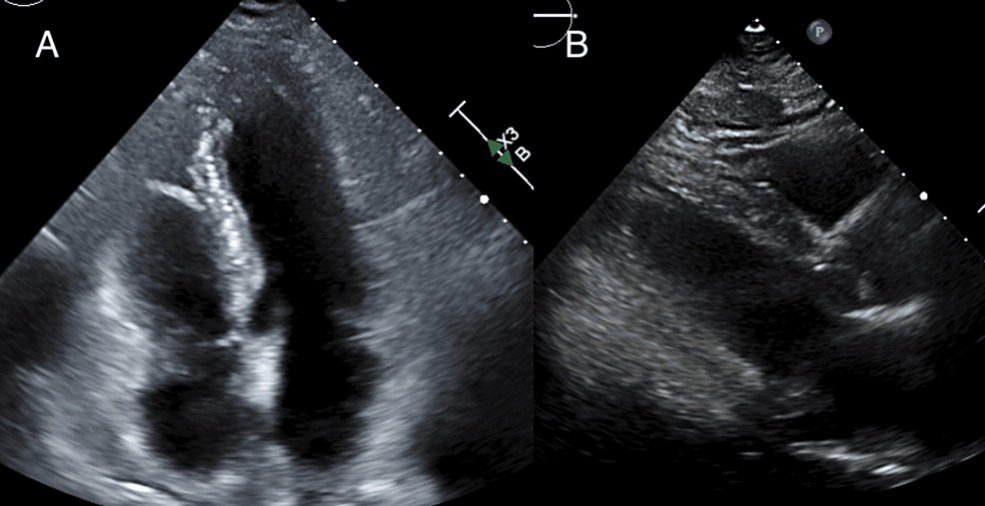
Echocardiogram images The ventricular and septal wall thickness appeared to be normal on the apical four-chamber view (A) and parasternal long-axis view (B).

During his hospitalization, an echocardiogram revealed a normal ejection fraction without structural or wall motion abnormalities (Figure 2). A nuclear stress test did not show ischemic changes, and his troponin I level returned to normal range within six hours. A 24-hour continuous telemetry and a following two-week ambulatory Holter monitoring showed occasional premature ventricular contractions with no clinical significance. There was no recorded arrhythmia or reported palpitation. Notably, the patient’s screening coronary artery calcium (CAC) score one year before this admission was 0.

Diagnosis, differential diagnoses, and management

The patient’s initial diagnosis was syncope, which was characterized by transient cerebral blood flow insufficiency and oxygenation inadequacy. Critical differential diagnoses of nonsyncopal transient loss of consciousness (TLOC) at the first encounter included cerebrovascular accident (CVA), seizures, acute coronary syndrome, etc. There was no neurologic focal deficit to suggest a CVA. The patient’s lack of postictal state or typical tonic/clonic movement rendered an epileptic disorder unlikely. Neither did his presentations suggest other possible explanations for his TLOC such as states with fluctuating vigilance, intoxication, psychiatric, or post-traumatic disorders.

The patient’s transient chest pain en route to the hospital was intriguing. His chest pain fell into the atypical angina/chest pain category, which could be suggestive of cardiac ischemia. The mildly elevated cardiac enzyme, together with ischemic symptoms and the absence of acute electrocardiographic ischemic changes, indicated a diagnosis of type 2 myocardial infarction (MI) [5]. Acute coronary syndrome (ACS), which is a diagnosis mutually exclusive to type 2 MI and represents a true acute coronary plaque event, was ruled out because of his benign ECG findings, short-lived chest pain, and quickly resolved cardiac enzymes.

The patient’s admission diagnoses were syncope and type 2 MI (also commonly referred to as “demand ischemia”). The initial treatment in the ED included one dose of aspirin 325 mg, atorvastatin 40 mg, potassium replacement, and intravenous normal saline infusion. The main purpose of his hospitalization was to investigate the underlying causes of his syncope. Syncope can be categorized into cardiac, reflex, and orthostatic etiologies [6]. Our patient's extensive cardiac investigations effectively ruled out acute coronary syndrome or structural heart disease, and no evidence of significant arrhythmia was found. His history excluded any prodrome or potential trigger of reflex syncope, such as vasovagal, carotid sinus-related, or situational syncope. Orthostatic syncope can be caused by a wide range of etiologies, including volume depletion, autonomic deficit, or as a side effect of certain medications. The patient had a euvolemic appearance with no underlying condition, such as diabetes or Parkinson’s disease, that could predispose him to autonomic failure.

The typical flushing that the patient experienced before losing consciousness and the robust chronologic relation between niacin intake and syncope suggested a drug-induced pathophysiology. Flushing typically commences 15 to 120 minutes after ingesting niacin, coinciding with the timing of the symptom onset in this patient [7]. Similar to the inadequate cerebral perfusion, a transient mismatch in demand and coronary blood supply explained his chest discomfort and transient myocardial injury leading to troponin elevation. The patient was diagnosed with niacin-induced syncope and was counseled to discontinue daily niacin as a supplement.

The treatment of type 2 MI in the absence of known CAD lacks trial data or guidelines [5]. Given his benign lipid profile, recent CAC score of 0, and incidental nature of the cardiac ischemia event, no further antiplatelet or statins were prescribed during or after his hospital stay. After discharge, the patient returned to his daily activity without limitations and stopped taking the niacin supplement. At a three-month follow-up, he remained at his baseline health state with no recurrent syncope, lightheadedness, or flushing following niacin cessation.

## Discussion

Niacin is the generic name for nicotinic acid and other related derivatives, including nicotinamide and nicotinamide riboside. It is naturally present in a wide variety of foods. The recommended dietary allowance (RDA) for niacin is 14-18 mg daily in the adult population [8]. The average daily niacin intake of adults above 20 years old in the United States, according to the National Health and Nutrition Examination Survey (NHANES) 2017-2020 pre-pandemic data, was 25.8 mg with a standard error of 0.34 mg [9]. Thus, the dietary intake was adequate to meet the daily niacin requirements rendering the supplementations unnecessary. On the other hand, 10.3% [10] of individuals who consume niacin-containing dietary supplements have a daily intake higher than the tolerable upper intake level (UL) (niacin UL for adults 19 years and older: 35 mg/day) [8]. Some niacin-only products contain 500 mg (26 times higher than the RDA) or a higher dose per serving [11], which greatly surpasses the RDA or even the UL for niacin.

The lipid-modifying effects of niacin are well-known and frequently used in commercial advertisements. Several early randomized clinical trials (RCTs) proved the long-term mortality benefit of niacin [2]. The caveats, however, should be noted. These RCTs included only subjects with severe coronary artery diseases such as a positive history of myocardial infarction or coronary bypass surgery. Moreover, the niacin doses used in these trials were as high as 3.0-4.2 g per day. Therefore, caution should be taken when generalizing the conclusion to a population without established diagnoses of CAD and to the lower doses (still high compared to usual dietary needs) offered by OTC niacin supplements.

With the frequent use of statins as lipid-lowering agents, more contemporary studies report the lack of clinical benefit of niacin in patients taking statins. For example, the AIM-HIGH (atherothrombosis intervention in metabolic syndrome with low HDL/high triglycerides: impact on global health outcomes) trial included 3414 subjects with established cardiovascular disease and investigated the efficacy of niacin from 1500-2000 mg [12]. Another trial, the HPS2-THRIVE (Heart Protection Study 2-treatment of HDL to reduce the incidence of vascular events) study [13], enrolled 25,673 adults with vascular disease and compared the effects of 2 g of extended-release niacin with 40 mg of laropiprant (a selective DP1 receptor antagonist inhibiting the flushing side effect of niacin) to placebo. Participants in both trials received background statin-based LDL cholesterol-lowering therapies. Interestingly, niacin did not show incremental cardiovascular benefit in either study [12,13].

The safety and toxicity profiles of niacin are rigorously characterized [14]. Skin flushing is the most common side effect of higher doses (0.25-3.0 g/day) of niacin treatment with a frequency of 30-92% [15]. Other recognized side effects include gastrointestinal discomfort, hyperglycemia, and hepatic toxicity. The vasodilatory effects of niacin that cause flushing and transient hypotension are mediated by an interaction between niacin and GPR109A receptors on the epidermal Langerhans cell, increasing the release of prostaglandin (PG) D2 into the systemic circulation [16,17]. However, the niacin-induced syncope or “fainting” was rarely described [4]. Tolerance to niacin can be achieved by either a prolonged course, using an extended-release formula instead of immediate-release ones, prophylactic aspirin or non-steroidal anti-inflammatory drugs (NSAIDs) [15], or adding laropiprant. Conversely, a hot bath, concomitant alcohol intake, or abrupt increase in dosage poses a higher risk of hemodynamic compromise causing syncope in extreme cases, as was presented in our patient.

It is clear that in this case of niacin-induced syncope, niacin should be discontinued due to the severe side effects. In general, how should physicians counsel their patients on the utility of OTC niacin-containing products? As a nutritional supplement, most individuals get sufficient niacin from foods and beverages. Many people who take dietary niacin supplements have unsafe daily niacin intake higher than the UL. For lipid-modifying purposes, high-dose niacin has no additional cardiovascular benefit while carrying the risk of adverse reactions. Niacin is no longer recommended by the current AHA/ACC guidelines on the management of blood cholesterol [18]. Additionally, many OTC niacin-containing product provides a dosage of niacin much lower than what was sufficient for a significant lipid-modifying result. In conclusion, dietary niacin supplements should be considered to have net clinical harm [19], and the risk of their regular use should be cautiously counseled or even discouraged.

## Conclusions

Niacin-induced syncope, albeit rare, can occur with an abrupt niacin dosage increase. This pathophysiology should remain in the differential diagnosis when encountering patients with syncope who take niacin supplementation. For patients who seek advice on niacin supplementations, a primary care provider may offer counsel that most people get adequate niacin intake from foods and beverages rendering extra supplementation unnecessary. Unsafe daily niacin intake exceeding the tolerable upper intake level (35 mg/day) is common in people who consume niacin-containing dietary supplements. Niacin exhibits no incremental cardiovascular risk reduction in the contemporary statin era and has no role in the primary prevention of cardiovascular disease. Regular dietary niacin supplements should be discouraged due to their net clinical harm.
